# Exploring Use, Usability, and Functionality in the Iterative Testing of a Co-Designed Preventative Mental Health Smartphone App for University Students: Comparison of Monash Thrive App Development Trials

**DOI:** 10.2196/67818

**Published:** 2026-11-12

**Authors:** Melinda McCabe, Michelle Byrne, Judith Gullifer, Isabelle Smart, Chunlei Chang, Sanket Singh, Gagan Khara, Kim Cornish

**Affiliations:** 1 Turner Institute for Brain and Mental Health, School of Psychological Sciences, Monash University, Level 5, 18 Innovation Walk, Clayton, Victoria, 3800, Australia; 2 School of Psychological Sciences, Monash University, Clayton, Victoria, Australia

**Keywords:** mental health, prevention, smartphone app, digital tool, university student, usability, iteration, design, co-design, systems, development

## Abstract

**Background:**

The emergence of digital tools in physical and mental health spaces is a necessary development to meet the growing need for preventative recognition and early care. Such tools must be appropriately tested throughout the development process to ensure that they are fit for purpose and both empirically and clinically valid. However, it is also critical that these tools are developed in an iterative and user-focused manner to ensure that they meet user needs for usability and functionality.

**Objective:**

This study explores the iterative testing of use, usability, and functionality key performance indicators of the Monash Thrive app, a preventative mental health app focusing on mental health literacy and mood check-in features. It was hypothesized that there would be an improvement in all variables in the second iteration testing of the app compared to the initial iteration.

**Methods:**

Two iterative trials of the app were compared, including an initial trial of the minimal viable product (MVP) of the app across 4 weeks of use and a secondary trial of the beta version of the app, redeveloped post-MVP testing, across 12 weeks of use. All recruitment and testing were delivered online through email surveys.

**Results:**

Retention rates for both trial 1 and 2 were low (59% and 46%, respectively). Despite redevelopment and resulting hypotheses, there was no significant improvement observed across any variable when comparing the 2 trials. No difference was identified across any Mobile App Rating Scale subscales, and a significant difference was detected (*P*=.04; 95% CI −0.59 to 9.35), indicating higher usability scale scores in the MVP app trial compared to the beta.

**Conclusions:**

Results did not support the study hypotheses. Such findings could be due to changing expectations of smartphone apps, relatively low retention throughout the trials, or the lack of a control or comparison group for which to compare ratings. However, these lack of findings demonstrate the core importance of iterative testing to ensure that changes or upgrades are delivering the desired improvement in use, usability, and functionality.

## Introduction

### Digital Tools in the Mental Health Space

Digital health tools including websites, smartphone apps, telehealth services, and other online or digitally facilitated services have become critical in the evolution of mental health care globally. Recent systematic reviews have highlighted the emergence and testing of digital health tools used in areas such as substance use disorders, mood and trauma-related disorders, and a wide range of other mental health conditions [1]. Although still a new and emerging technology in the mental health space, websites, apps, and other digital tools are the clear future steps forward to assist in the availability, accessibility, and scalability of mental health support. These benefits are clear in preventative mental health care, which describes the digital tools, specifically websites and apps, targeting and tailored to individuals who may be struggling with their mental health and well-being without having or necessarily meeting the criteria for a mental health diagnosis. Preventative tools and care can be designed for and deployed among the broader population with the overarching goal of promoting awareness, recognition, and early help-seeking well before clinical symptoms emerge. Many such apps have been developed and tested in a clinical and research setting, focusing on preventative care or support care [2-9]. Although there are differing levels of efficacy and effectiveness across these tools depending on core outcomes and use rates, almost all of these apps are either not available for widespread use or have been removed from relevant app stores. In the mental health space, efficacy is critical and requires a strict testing protocol to ensure that users are capable of benefiting from the tool. However, this is not enough to ensure that these tools are available for ongoing use and can evolve to meet the needs of the users over time.

### Efficacy and Usability

Efficacy of preventative mental health tools, particularly those tools developed from a clinical or research-based setting, is often measured during development through structured research testing. In the mental health space, such testing often uses clinically validated measures of stress, well-being, and other measures to assess whether use of the app has caused a measurable change in mental health symptoms after a period of use [4-14]. Although such scales and the focus on mental health outcomes are necessary to ensure that the digital tool being developed improves the health and mental health outcomes being targeted, efficacy measured through mental health outcomes is not able to account for elements critical to uptake and use. Usability must also be a core focus of tool testing and design, specifically usability in terms of ease of use and how intuitive the tool is. Usability also includes the burden or time cost of the user to effectively engage with the tool (ie, how long does it take to learn how to use) and the ability of the tool to effectively deliver on its intended functionality. Such elements of design and delivery are critical to ensure the uptake and ongoing use of the app required to make sure that the intended user base interacts with the tool long enough to see mental health improvements outside the confines of clinical trial or research settings.

### Including Critical Design Stages in Research

The focus on usability is a well-established and accepted foundation of product design in industry. The importance of usability, to ensure a product can be used effectively and efficiently by the target user, can be the difference between the success or failure of any new product or smartphone app, regardless of how effective the tool may be. In health and mental health spaces, the focus on usability can be overlooked or seen as secondary to the objective efficacy or evidence base of a product; however, if the use of the tool is dependent on a user-driven uptake and ongoing use, it is critical that the usability, engagement, functionality, and efficacy are all considered key performance indicators. Such indicators can be measured and identified by specific tools including the System Usability Scale (SUS) [15-18] and the Mobile App Rating Scale (MARS) [19-25]. These scales demonstrate how distinct and interconnected each of these usability measures is. The MARS contains 5 subscales, exploring a wide range of elements contributing to app quality. These subscales include engagement (how entertaining and interesting the app is), functionality (performance and ease of use), aesthetics (quality of layout and graphics), information (quality and quantity of information), and subjective quality (user use, recommendations, or desire to pay for use). The SUS is a validated scale that can be used to assess a wide variety of tools, not just smartphone apps. Originally developed in 1996 [15], this tool is still relevant, exploring whether participants would use the tool regularly, if they would need support or training to use the tool, and if they felt confident using and navigating the system or tool. Improving usability is an ongoing process that takes place in both initial and ongoing development phases. Such development processes require iterative stages of product development that rely upon user testing and feedback throughout the development process, including ongoing testing with final release products to ensure a product evolution that can adapt to meet user needs. Many theories and processes exist to outline these necessary steps; however, one of the most recognizable in literature is the user-centered systems design (UCSD) [26]. The UCSD process highlights the importance of an agile, user-focused, and iterative design process. Using an iterative and circular structure of (1) vision and plan, (2) analyze user needs, (3) design for usability with prototypes, (4) construct and deploy in iterations, (5) evaluate with users in context, and (6) feedback and integration into the next iteration, cycling back into stage 1 to repeat the process. Two of the greatest challenges throughout this process are the ongoing iterative testing and development and ensuring the focus on user-focused testing and the importance of user feedback. Iterative testing and subsequent redevelopment require substantial time and financial commitments, often better used in environments that have ongoing funding and continuous development goals. This may be why iteration is often given in commercial settings, but can often be underused in academic or health settings where funding may be limited by short-term grants. Iterative testing and development is not a new concept in literature and has been used in app testing [27,28]; however, the use and reporting of iterative testing in academia are inconsistent, where some studies refer to iterative testing as stages of testing of the same tool from different perspectives [3,29]. A focus on the end user during development is more common and is continuing to grow as a new standard for intervention testing and can be referred to as co-design or consultation. Some inconsistency remains when defining how end users are included in the design process.

### Potential to Develop Engaging Tools Using Evidence

The term “co-design” can mean many things in the fields of research, development, and health services [30,31]. For many research-based and academic-focused teams working on a co-design process, this means the consultation and feedback gathering from experts in the field, such as qualified health care providers or implementation experts or those with lived experience such as caregivers or advocates. Although consultation with these expert bodies is important, particularly in a health setting, it does not consider the needs of the individual user from the experience of the individual user. This means the co-design and design consultation happening in these academic or clinical settings can bias the opinions of a small number of experts rather than potential users who are themselves experts in lived experience and are better equipped to inform developers about their own desired functionality or barriers to use. It is this information that can be critical to user uptake, endorsement, and ongoing use. In a broader industry setting, outside the world of academia, co-design is the focus and importance of end-user consultation at iterative stages of development to inform and guide planning and final decision-making for the end product. The landscape of how these digital tools are being developed in medical and academic settings is evolving with more studies emerging, demonstrating both user-focused co-design and iterative design process [32,33]. A core foundation of these steps toward the future of health and mental health digital tool development in academic and medical settings is the incorporation of scales and key performance indicators, such as those measured by MARS or SUS, that focus solely on the user experience, engagement, and likelihood of further use and success of the tool, in addition to the health or clinical scales used to measure changes in participants over time. The inclusion of scales such as the MARS and SUS will ensure that researchers alongside developers are focusing on both the efficacy of the tool in terms of their intervention of interest (mental health outcomes) and also whether the tool is a usable, engaging, and effective way of delivering their interventions. It is critical to ensure that not only is a health or mental health tool founded in strong evidence, but that it is also usable to the degree that an individual user would willingly use the tool long enough to see benefit outside the extrinsic motivators of a research trial, clinical oversight, or other kind of incentive.

### This Research

The iterative testing of product usability and functionality is a fundamental element of all product development and testing. In the digital mental health space, app testing often focuses on mental health and health-related outcomes, focusing on clinical or research efficacy. The inclusion of design and usability-focused measures is slowly being incorporated into such development and testing; however, the importance of iterative testing is still underreported in the literature. The Monash Thrive app is a preventative mental health app designed and developed by a team of both mental health researchers and educators and experts in user experience and systems design. The design process of the Monash Thrive app followed the iterative and user-focused steps highlighted in the UCSD process. The focus of the app is to promote mental health awareness and literacy, the early recognition of mental health concerns, and early help-seeking behaviors of university students. The Monash Thrive app was designed to deliver a range of mental health literacy tools and promote preventative mental health behaviors such as journaling [34] and mindfulness [35]. At the time of development (2020‐2022), no single app or tool was freely available to university students, which allowed users to test and incorporate a range of positive mental health tools. The aim of this study is to explore the user-focused testing, design, and usability key performance indicators of a preventative mental health app designed with and for a university student population across 2 iterative app testing trials using both the initial minimum viable product (MVP) and the second iteration (beta) app prototypes. It is hypothesized that the trial of the second iteration of the Monash Thrive app (beta) will show improvements in all use, usability, and functionality outcome variables when compared to the initial iteration and trial (MVP) of the Monash Thrive app.

## Methods

### Participants

#### Trial 1

Participants were recruited using an online recruitment and eligibility survey that was open for a period of 13 weeks. This survey included an explanatory statement and consent form. All eligible participants who had responded to this survey by the close date were recruited and provided with details to download and install the Monash Thrive app. All respondents who met the eligibility criteria and responded before the start deadline were then recruited. The inclusion criteria for trial 1 required participants to be currently enrolled at Monash University at the time of participation, to be located in Australia due to app accessibility, and to have a working smartphone. Surveys including items not included in this study, app use, feedback, and bug reporting were sent to participants weekly. The items of interest in this study were included in the week 4 (of 4) surveys. Initially, 135 participants were recruited throughout the 4 weeks of active app use and 6 weeks of survey testing of trial 1. From the initial 135 participants, only 80 completed the survey containing the core items of interest; 1 participant did not complete all items of interest on the MARS and did not complete the SUS. A total of 79 (59%) participants completed all items of interest in this study and are therefore included in the following analysis. All 79 participants were located in Australia, Victoria, at the time of testing. Approximately 70% (55/79) of these participants were international students, 79% (62/79) identified as female, and 58% (46/79) of the sample were in their first year of study. The average age of the participants was 21 (SD 4.96) years, and the age range of the final sample was 18 to 50 years. Across users, 63% (50/79) of participants used an iPhone, and the remaining participants used a range of Android devices including Samsung, OnePlus, and Redmi.

#### Trial 2

Similar to trial 1, an online recruitment and eligibility survey (including explanatory statement and consent form) was open for 11 weeks, and all eligible participants were contacted after survey close with details to download and install the Monash Thrive app. Participants from trial 1 who indicated that they would be interested in taking part in another trial were also contacted with an invitation to complete the eligibility survey. Similar to trial 1, all respondents to the eligibility survey who met the inclusion criteria were recruited and onboarded. Trial 2 included 5 waves of recruitment, where new responses to the onboarding survey were included in the next wave of recruitment until the deadline. The same inclusion criteria were used as in trial 1; however, for the final analysis, those who completed trial 1 were removed from the trial 2 data in this study. New surveys were delivered to participants fortnightly, measuring nonreported items of interest and app use, feedback, and bug reporting. The items of interest in this study were included in the week 10 (of 12) surveys. Initial recruitment for trial 2 included 100 participants; by the end of the trial, 46 participants completed all items of interest across the 12 weeks of testing. From the initial 100 participants, only 49 participants completed the survey including the 2 core items of interest; of these 49, 3 did not complete all the items of MARS. Of the 46 (46%) remaining participants included in the final analysis for trial 2, 74% identified as female, 24% as male, and the remaining 2% did not identify with the listed options. Of the final sample, 38 were international students (83%), and just under half (46%) were in their first year of study at the time of the trial, and all but 1 participant was located in Australia at the time of recruitment. Of the final sample for trial 2, 59% used an Apple smartphone, and the remaining used a variety of Android devices. The average age of the sample was 23 (SD 5.86) years.

### Measures

#### Monash Thrive App

The Monash Thrive app is a co-designed preventative mental health app that has been developed, tested, and deployed by a team of both mental health researchers and educators, alongside expert app developers and designers to ensure that it met both the expectations of the market, users, and industry, and was able to meet the efficacy standards of empirical testing. The app focuses on increasing the users’ personal awareness of not only their own mental health, but also their understanding of mental health and psychology as a field. The overarching goal of the app is to improve awareness and increase rates of early help-seeking for mental health concerns well before the onset of clinically significant symptoms.

The first functional iteration of the Monash Thrive app, the MVP, was tested among participants in trial 1. The core functionality of the MVP was based on a multistage mood check-in including a diary entry. Based on users’ “check-ins,” they were then recommended mental health literacy resources as a short-form “blog” that can be read by either flipping through a series of cards or in long form. The entire series of resources was also available for users to explore. Alongside these interactive tools, the MVP also included a character “buddy,” Moody, who sent reminders and responded to mood check-ins. The Monash Thrive app also included reminders to engage with the app; these were set at 9 AM and 6 PM daily, and these reminders could be blocked using phone settings. The MVP iteration of the Monash Thrive app was designed and built based on a combination of market research focusing on the core functionality of the most popular mental health and well-being focus smartphone apps and informal community-based interviewing where the lead designers and developers engaged with students in community settings to informally discuss app use and desired characteristics and features. From this, the core elements of a mood check-in and accompanying student-written mental health resources (peer-developed) formed the basis of the Monash Thrive app and therefore the minimal viable necessary features. The second iteration of the app, tested in the second trial, included all the same functionality as the MVP with the addition of a personalized analytics page and calendar function that allowed users to reflect on their mood check-ins over time. The second iteration of the app also allowed users to customize or change reminder notifications in the app. New developments were based on user feedback from both the qualitative and quantitative survey data and informal user focus groups via Zoom (Zoom Video Communications) data collected in trial 1. Example screenshots of the described functionality can be found in Multimedia Appendix 1. User feedback and market research formed the basis for feature planning and development. Throughout both trials, the Monash Thrive app was only available for download for participants of each trial through software testing platforms (eg, TestFlight), and the app was not available publicly through app stores.

#### App Quality

The MARS [19] was used to measure the quality and functionality of the first and second iterations of the Monash Thrive app. The MARS is an extensive 5-point Likert scale with 29 items, exploring engagement (5 items), functionality (4 items), aesthetics (3 items), information (7 items), subjective quality (4 items), and an app-specific score (6 items), as well as a total score. All items have individually coded responses ranging from 1=negative to 5=positive. The MARS also includes 6 items focusing on app-specific perceived impact of the digital tool, and these items are tailored to the app in question and are answered using a 5-point Likert scale of 1=strongly disagree to 5=strongly agree. The MARS is scored by calculating the mean score across all items within each subsection of the scale. Initial development of the MARS app demonstrated strong reliability (α=.90) [19]. For this study, 4 items were removed from the MARS total scale (accuracy, goals, visual information, and evidence base), as they were not relevant to the initial version of the app being tested. Therefore, the information subscore and total score will not include these 4 items and will be calculated using the average of the remaining items. The reliability of the MARS items (total) used in this study was α=.93 for trial 1 and α=.92 for trial 2.

#### Usability

The SUS [15] was used to measure the usability of the Monash Thrive app in trial 1 and trial 2. The SUS comprises 10 items scored on a 5-point Likert scale of 0=strongly disagree to 4=strongly agree. All even-numbered items are negatively worded and are therefore reverse-scored before all items are summated and multiplied by 2.5 to allow for a final possible score range of 0‐100. Previous analysis of digital health apps demonstrated a benchmark SUS score of 68 (SD 12.5), which could be considered a “good” score [36]. A 2018 study found that the SUS was still being used by up to 43% of industry usability studies [17] and has consistently been cited as a scale easily understood by a variety of users (researchers, developers, users, and project managers) [37].

#### App Use

The Monash Thrive app was designed to not collect and store user data, including automatically collected use and engagement analytics. This was to ensure complete privacy and safety of user mental health–related information such as mood and emotion check-ins and diary entries. As such, to measure app use, a short series of self-report items was created by researchers to assess average use in minutes over the course of the previous 7 days to measure and track the average time (minutes per day) spent using the app over the course of both trials. These items listed the day and date of the previous 7 days and provided participants with a sliding scale from 0 to 60 minutes each day in which to respond. Participants were instructed that if the app was used for more than 60 minutes to just indicate 60 minutes of use. Given the anticipated time of use per day was between 5 and 10 minutes, researchers did not anticipate any issues caused by a reported use ceiling. Participant average scores were calculated as the average of at least 3 inputs from participants. If participants did not input at least 3 responses, they did not have an average use score and were therefore not included in the relevant analysis.

### Procedure

Trial 1 included 6 weeks of testing across 4 weeks of app use (4-week surveys followed by 2 weeks of informal focus group testing). Expression of interest, mental health variables, and demographics were collected prior to app use. Based on these initial responses, participants were then invited to participate and guided through the download and installation process by using step-by-step manuals specially designed for iOS and Android devices. Once onboarding was complete, participants received a follow-up email each week containing surveys on use and app feedback (each week), mental health outcomes (weeks 2 and 4), and app quality and usability (week 4). This was then followed by informal focus groups to further explore user feedback through open discussion via Zoom. The design of these focus groups followed a market research model, and due to the unstructured nature of the discussion, it was not included in this analysis. Trial 2 included 12 weeks of testing and app use, with demographic, onboarding, and baseline mental health variables collected prior to testing. During the testing period, participants were surveyed every 2 weeks with use and app feedback (weeks 2, 4, 6, 8, 10, and 12), mental health battery (weeks 6 and 12), and app quality and usability (week 10). All surveys included a reminder for any participant who had not completed the survey after 1 week. All surveys and reminders were self-reported measures and sent to participants via email and completed online in their own time. Convenience sampling was used across both trials 1 and 2, and advertisements were used to invite participants to participate, both online and in targeted student locations. Participants in trial 1 were also invited to take part in trial 2 if they opted in for recontact. No participants who took part in trial 1 were included in the findings for trial 2.

### Ethical Considerations

Across both trials, participants were reimbursed with an online gift card sent at the end of the trial. For each survey the participants completed, they added Aus $10 to the final amount they would be reimbursed upon the conclusion of the trial. Participants in trial 1 were also reimbursed an additional Aus $20 for participating in the informal focus groups. Participant payments for trial 1 were completed in May 2022, where the average exchange rate from the Australian dollar to the US dollar was US $0.705. Payments for trial 2 were completed in December 2022, where the average exchange rate from the Australian dollar to the US dollar was US $0.6745. Participation in both trials was voluntary and provided a full explanation of the study via explanatory statements and informed consent as part of the recruitment survey. Any and all potentially identifiable data were removed from this data analysis or displayed in an aggregate form to ensure the privacy of the participants. Both trial 1 (ID# 30475) and trial 2 (ID# 32761) were approved by the Monash University Human Ethics Committee. Both trial 1 (ACTRN12622001054707) and trial 2 (ACTRN12623000131651) were registered with the Australian and New Zealand Clinical Trials Registry prior to testing.

## Results

All data analysis was completed using SPSS Statistics (IBM Corp). A series of 1-tailed *t* test analyses (α=.05) was used to assess the differences between the app design and quality across trial 1 and trial 2. Normality was assumed based on kurtosis and skewness variables (<1.96) aside from the average time of use (kurtosis=2.28). No participants who were included in the sample of trial 1 were also included in trial 2. Homogeneity of variances was assumed for all variables aside from MARS functionality and MARS aesthetics based on the Levene test for equality of variances (*P*>.05). As such, average time of use, MARS functionality, and MARS aesthetics were analyzed using the Welch *t* test [38].

Descriptive statistics for all analyses can be found in Table 1. All inferential statistics can be found in Table 2.

For all variables, significance measured via *P* values was only reported for app usability when comparing both trials. However, this difference was in a direction opposite to what was hypothesized, and as such, the null hypothesis was retained. This finding is also supported by the reported CI.

**Table 1.** Descriptive statistics.

	Trial 1 (n=79), mean (SD)	Trial 2 (n=49), mean (SD)
App use (minutes)	5.59 (2.91)	4.94 (3.82)
Engagement	3.44 (0.62)	3.44 (0.62)
Functionality	4.23 (0.61)	4.17 (0.53)
Aesthetics	4.17 (0.65)	4.14 (0.53)
Information	3.88 (0.72)	3.93 (0.64)
Subjective quality	2.91 (0.67)	3.03 (0.74)
App utility	3.88 (0.73)	3.97 (0.60)
MARS^a^ total score	3.73 (0.50)	3.76 (0.47)
App usability (SUS^b^)	79.23 (12.85)	74.89 (15.45)

a MARS: Mobile App Rating Scale.

b SUS: System Usability Scale.

**Table 2.** One-tailed *t* test outcome statistics across trials.

	95% CI	*t* test (*df*)	*P* value
App use (minutes)	−0.94 to 1.05	0.10 (192)	.46
Engagement	−0.27 to 0.17	−0.45 (127)	.33
Functionality	−0.16 to 0.24	0.40 (114.93)	.35
Aesthetics	−0.18 to 0.22	0.19 (115.77)	.43
Information	−0.29 to 0.21	−0.34 (126)	.37
Subjective quality	−0.37 to 0.12	−1.0 (126)	.16
App utility	−0.34 to 0.13	1.74 (126)	.18
MARS^a^ total score	−0.22 to 0.14	−0.46 (123)	.32
App usability (SUS^b^)	−0.59 to 9.35	1.74 (126)	.04

a MARS: Mobile App Rating Scale.

b SUS: System Usability Scale.

## Discussion

This study explored design and functionality key performance indicators of a preventative mental health app (Monash Thrive app) across 2 stages of iterative testing, an MVP trial and a beta trial after a redesign and development process based on the MVP testing. Key variables of interest across this study included average app use, system usability, and various functionality and perceived app quality markers outlined by the MARS including engagement, functionality, aesthetics, quality of information, subjective quality, and app utility. When comparing app system usability and functionality markers across trials, a significant difference was only identified for app usability across trials; however, this significant difference demonstrated a decline in system usability across trials, the opposite of what was predicted in the study hypotheses.

Although there was no improvement across trials, the average scores of the MARS and the MARS individual domains for both trials indicate a positive response for each of the items within each domain. The strongest domains for both trials were app functionality, exploring how accurate, quick, easy to learn, and intuitive the app is, and app aesthetics, exploring size and arrangement of visual elements, resolution and quality of visual elements, and how subjectively “good” the app looks. This was followed by the quality of information provided within the app (information), app utility and efficacy based on target outcomes (app utility), how entertaining, interesting, customizable, and engaging the app is (engagement), and finally, subjective rating of the app and recommendation to others (subjective app quality). These scores are lower than similar ratings of health apps completed by researchers or health experts [22,39-43]; however, there is much fluctuation within these published average scores (ranging from 3.08 to 4.2 for research reporting mean total scores). Understanding how these scores relate to other studies that used the scale with a user-based sample is limited.

The lack of change in average scores across time for all MARS variables does not support the previously outlined hypotheses. These findings do highlight the critical importance of ongoing testing throughout the iterative development stages, where initial findings in the first app trial indicated strong scores; however, the implementation of suggested changes and retesting of the beta iteration of the app demonstrated similar average scores on the MARS variables. Without iterative testing, developers may infer that changes based on user feedback must improve all design outcomes; however, without ongoing testing, this cannot be assumed.

Similar to the findings with MARS outcomes, the usability score from the SUS demonstrates strong results both in trial 1 and trial 2 when compared to similar health app testing [36]. When initially comparing the 2 trials directly, there was a significant difference between the 2; however, this was not supported by the accompanying CI outcomes of the same test, and the difference was in the opposite direction to what was hypothesized. Much like the findings for the MARS, these unanticipated differences are evidence that the ongoing testing of iterations throughout development is critical to understand how changes, even if recommended by users in previous testing, may not improve system usability. In this study, the changes across app iterations increased functionality, with the introduction of personalized mood analytics and additional steps available to review mood check-ins over time through the new calendar view function. These additional features, though based on feedback from users, may have increased the complexity of the app without the inclusion of necessary changes to ensure that engaging with these new features is intuitive to users. These findings may have also been impacted by ever-changing expectations and rising standards of digital tools, a greater expectation of a tool known to be the “second iteration” of a tool, or high expectations from a tool associated with their university. Although no users in trial 1 also responded in trial 2, it is possible that expectations of mental health digital tools evolved between the 2 trials.

Across both trials, the average daily use of the app, reported by participants, was approximately 5 minutes (trial 1: mean 6, SD 3 minutes; trial 2: mean 5, SD 3 minutes). This reported use matches the intended daily use of the app, where daily interactions should consist of a mood check and engaging with a mental health resource based on their mood check-in. The beta app also provides users the ability to review their own mood check-ins over time; however, this was unlikely to add much time to the interactions.

This study, as a comparison of 2 iterative trials, is an example of the importance of iterative testing across key design and performance indicators. Although iteration is not uncommon in an app design process, it is not often reported or explored through literature in the same manner as we might see single trial or mental health outcomes tested. By systematically comparing all variables of interest across app iterations, developers can ensure that changes and new developments are improving the app from the user’s perspective and experience. Although these findings represent one of the first empirical comparisons of outcomes across iterations of the same app or tool, there are limitations in the design and interpretation of these findings. The varying length of each trial and the difference in time frame are worth noting when interpreting the findings. Trial 1 extended over a 4-week period of active app use, where the items of interest were collected after 4 weeks of use, and trial 2 extended over 12 weeks of use, where the items of interest were collected at 10 weeks of use. Although not previously highlighted in any of the scales used in this study, there may have been some bias and changes in user response due to the differences in time spent using the app. Both trials could have been improved by the use of a more structured trial protocol such as the inclusion of a control group, blinding across groups, the inclusion of another intervention for comparison (eg, a nondigital intervention or another popular app), and the collection of additional relevant demographic information such as digital literacy, typical phone use, and socioeconomic status. A high number of international students when comparing trial 1 and trial 2 should also be noted (70% and 83%, respectively); this may be due to the advertisement of the Monash Thrive app testing trials, where promotion on site and online was more heavily promoted in international student spaces. This skew toward international students may impact findings if participants experienced any language or cultural barriers to engaging in the mental health content of the app that was primarily built from Western research and principles. Future research may consider the inclusion of more culture-focused survey items such as qualitative open-ended questions to better understand how their digital tool may be interpreted and endorsed across a range of cultural and linguistic backgrounds. This study also demonstrates a significant and likely influential attrition rate of participants over time, where 42% and 46% of participants did not continue responding to surveys from trial 1 by week 4 and trial 2 by week 10, respectively. Although attrition is not uncommon in app testing trials [2-9], the drop in participation over time is critical to consider when discussing the usability of an app, where the discontinued completion of surveys likely reflects a discontinued use of the app despite the monetary incentive provided by each trial. Future studies may consider including attrition as a measure itself to reflect usability and continued use. Future studies may also benefit from the incorporation of objective measures of use and engagement. The app tested in this study was designed to not collect and send user data to protect user privacy; as such, all outcomes were collected through separate surveys including daily use, which required self-reporting and was collected once a week (for each day in the previous week) in trial 1 and once every 2 weeks (for each day in the previous 2 weeks) in trial 2. This does leave considerable margin for error in participant recollection and self-reporting of their use. Overall, any replication or similar research should undergo considerable improvement in trial structure, similar to that of randomized controlled trials.

In conclusion, this study is an example of the importance of incorporating design-, usability-, and functionality-based key performance indicators to app testing in a research and clinical setting. These outcomes provide the insight necessary to ensure that research-based and clinical digital tools in both the health and mental health space are not only found in clinical evidence but also usable and engaging enough for users to ensure the uptake and ongoing use necessary to see benefits. This study also highlights the importance of iterative testing throughout the design process, where developers may assume that the next iteration of design would demonstrate better usability and functionality scores; however, current findings demonstrate that even when changes are based on user feedback and are designed to increase app usability, such improvements may not be reflected in user testing. Iterative testing is important to ensure that app development is moving in the right direction and developers are not just assuming improvement in app usability and reception alongside the addition or changes in the future. This process is the foundation of a user-centered design process [26], which outlines the importance of continued testing and feedback incorporation as the tool evolves. The process of developing and testing these mental health and health digital tools is time-consuming and costly; however, none so more costly than the final development and deployment of a tool just to see low uptake and poor retention rates, resulting often in the removal of the app from circulation; as such, it is critically important that all relevant key performance indicators are included in testing of digital tools, iteratively, as they are being developed.

## Supplementary material

10.2196/67818 Multimedia Appendix 1 Screenshot images of the core functionality of the Monash Thrive app including (A) the home page with app character “Moody” and recommended mental health resources, (B) mood check known as “wheel of feels,” (C) second stage mood check-in including journal entry, (D) mental health resources library, (E) example of a mental health resource including short form (card swipe) and long form below, and (F) personalized mood analytics with calendar diary icon to review mood check-in timeline (available in app beta, trial 2).

## Abbreviations

MARS: Mobile App Rating Scale
MVP: minimum viable product
SUS: System Usability Scale
UCSD: user-centered systems design
