# Supplementary material for: Exploring Use, Usability, and Functionality in the Iterative Testing of a Co-Designed Preventative Mental Health Smartphone App for University Students: Comparison of Monash Thrive App Development Trials
Source: JMIR Hum Factors. 2026 Nov 12;13:e67818. doi: 10.2196/67818 (PMC13758967; doi:10.2196/67818)
Supplement: Supplementary file 1 [file humanfactors-v13-e67818-s001.docx]

**A.**

Screenshot images of the core functionality of the Monash Thrive app including the home page with app character ‘Moody’ and recommended mental health resources (A), Mood check known as ‘wheel of feels’ (B), second stage mood check in including journal entry (C), mental health resources library (D), example of a mental health resource including short form (card swipe) and long form below (E), and personalised mood analytics with calendar diary icon to review mood check in timeline (available in app Beta, trial 2) (F).
